# Three-Dimensional Motion Analysis of Finger in Sport Climbing in Different Age Groups

**DOI:** 10.3390/bioengineering13080865

**Published:** 2026-07-27

**Authors:** Julia Leipner, Gabriella Fischer, Michelle Bäbler, Peter Wolf, Andreas Schweizer, Lisa Reissner

**Affiliations:** 1 Department of Orthopedics, Balgrist University Hospital, 8008 Zurich, Switzerland; andreas.schweizer@balgrist.ch (A.S.); lisa.reissner@balgrist.ch (L.R.); 2 Division of Plastic Surgery and Hand Surgery, University Hospital Zurich, 8091 Zurich, Switzerland; gabriella.fischer@usz.ch; 3 Laboratory for Movement Biomechanics, Department of Health Sciences and Technology, ETH Zurich, 8092 Zurich, Switzerland; michelle.baebler@hest.ethz.ch; 4 Sensory-Motor Systems Lab, Department of Health Sciences and Technology, ETH Zurich, 8092 Zurich, Switzerland; peter.wolf@hest.ethz.ch

**Keywords:** climbing, kinematics, 3D analysis, hand biomechanics, injury and prevention, kinetics

## Abstract

Climbing with extreme finger positions is a risk factor for osteoarthritic changes. Our initial study revealed a large variation in PIP joint flexion, especially in older climbers. This study compares finger joint motion between age groups and examines relations to degenerative changes. Nine young (~33.9 years) and 11 old (~55.8 years) participants climbed a defined boulder problem in four grip positions: crimp, half-crimp, open-hand and participant-selected grip. Finger and wrist kinematics were recorded by an optical motion capture system, contact forces were measured using four instrumented holds, osteophytes were assessed using ultrasound and maximal finger strength was measured separately. Older climbers demonstrated significantly (*p* < 0.0001) reduced PIP flexion compared to younger climbers in crimp (65° vs. 85°), half-crimp (53° vs. 75°), and self-selected grip positions (56° vs. 76°), with no difference observed in open-hand grip (33° vs. 37°; *p* = 0.44), which is biomechanically less demanding on the PIP joints. The self-selected grip corresponded to the half-crimp position. Osteophytes were rare in younger climbers (PIP: 8.33%, DIP: 16.67%) but common in older climbers (PIP: 68.18%, DIP: 81.82%), with a negative correlation with PIP flexion and a positive correlation with DIP flexion without age differences in maximal force (limited to male climbers). The half-crimp position generated higher maximal finger strength than the open-hand grip, and maximal finger strength was applied with a more open hand position. Older climbers apply lower finger forces and demonstrate less extreme finger joint kinematics associated with higher osteophyte prevalence, while preserving maximal finger strength, suggesting adaptive, joint-protective movement patterns.

## 1. Introduction

Sport climbing and bouldering have rapidly grown in popularity, particularly since their Olympic debut in 2021 [1]. Increased participation has led to a rise in climbing-related injuries, which are typically classified as impact injuries, affecting the lower limbs, and non-impact or chronic overload injuries, mainly impacting the upper limbs and soft tissues [2,3,4]. The professionalization of the sport has shifted injury patterns toward chronic overuse, especially among young athletes exposed to high training volumes [3,5]. The most frequent finger injuries involve ruptures of the A2 and A4 flexor tendon pulleys, with the A2 pulley in the middle and ring fingers being particularly at risk [2,3,6,7]. Flexor tendon pulleys are essential for proper tendon guidance and force transmission in the hand. Rupture results in loss of tendon alignment and bowstringing, thereby compromising hand biomechanics [7,8,9,10].

Despite technique, the geometry of climbing holds influences finger positioning and joint loading. The main grip types—full crimp, half-crimp, and open-hand grip—differ in joint angles, contact area, and resultant forces, and thus likely impose distinct mechanical demands on both soft tissue and joint surfaces. On small edges, the crimp is typically the preferred technique associated with the maximal possible contact forces: only the tip of the distal palmar phalanx contacts the hold, the DIP joint is hyperextended, and the PIP joint is markedly flexed (~90°, with substantial inter-individual variability of ~60–100°, as observed in our prior work) [11]. The thumb is mostly placed over the index finger to generate higher contact forces, which increases loading of the flexor pulley system, tending to increase pulley injuries [2]. While the FDP transmits higher muscular forces in the crimp position, especially on small holds with half a size of the distal phalanx, the flexor digitorum superficialis (FDS) creates more strength on larger flat holds [12].

From a mechanical perspective, joints experience a tilting moment and a transition during such extreme finger positions that reduce articular congruency and produce focal, high-pressure contact areas, which have already been investigated in joints such as the hip, knee and shoulder [13,14,15,16]. Axial rotation of finger joints was observed in our initial study [11]. Also, Bärtschi et al. demonstrated that PIP joint translation increases under these conditions, making it vulnerable to periphyseal injuries in adolescent growth [17]. Several publications reported a bone reaction in the PIP and DIP joints due to mechanical load after several years of climbing, even in young subjects, without a correlation with pain [18,19,20,21,22,23]. Bollen et al. saw radiological clear signs of osteoarthritis in climbers over 40 years of age [20], and cortical bone changes were seen early, even in 10 years of follow-up compared to non-climbers [24]. In particular, the middle and ring fingers are under greater force and risk during climbing [22,25]. In contrast, the more common genetic-caused finger polyarthritis showed a different osteoarthritis clustering [26,27]. In the general population, the prevalence of hand osteoarthritis and radiographic osteophytes increases with advancing age and is generally higher in women than in men [28,29]. Epidemiological studies further demonstrate that the prevalence of radiographic hand osteoarthritis, including osteophyte formation, begins to increase during the fifth decade of life and continues to rise progressively with advancing age [30]. Therefore, both age- and sex-related differences should be considered when investigating osteophytic changes in climbers. These findings support the importance of further biomechanical understanding of grip configuration, joint angles, and load distribution on the articular surfaces of the finger joints, tendon and pulley system contributing to degenerative changes such as osteophyte formation in relation to age- and sex-related differences.

Our initial study [11] established the feasibility of using an optoelectronic motion capture system for the kinematic analysis of the finger joints and wrist during climbing. This pilot study enabled a first basic characterization of the joint angles and flexion-extension motions [31] for the common grip positions during climbing. We observed large inter-individual variation in PIP joint flexion and indications that older climbers exhibit reduced angulation across different grip positions. Due to the limited number of older climbers included in the previous study, the interpretation of the observed different joint angulations was limited, and age-related differences could not be assessed reliably. Having established the feasibility of using the optoelectronic motion capture system, we designed a more representative bouldering task that incorporated changes in foot position to better reflect actual climbing movements than the simplified protocol used in the previous study. Prior studies examining age- and climbing-related differences have primarily assessed adaptations of soft tissue and bone, rather than biomechanics or movement patterns during climbing [22,32,33].

This study aimed to compare finger joint motion between a larger age-stratified cohort of younger and older climbers as well as a more representative actual climbing task incorporating changes in foot position, taking into account the degenerative aging process of the finger joints.

## 2. Materials and Methods

### 2.1. Participants

Twenty-one healthy, experienced sport climbers participated in the study. To have a consistent climbing level to compare the data between the participants, we set the self-reported climbing level for red pointing (no fall or rest in a climbing route or boulder) in lead climbing on French-scale 8a or higher and in bouldering on Fontainebleau-scale (Font) 7b+ or higher. The international rock climbing research association (IRCRA) transformed all known climbing grades to a comparable scale for statistical analysis [34]. Corresponding to the IRCRA, we only included participants with a level of ≥23 IRCRA reporting scale (Table 1). Also, criteria for exclusion were inflamed joints or other illnesses that had an impact on the performance of climbing.

Participants were stratified a priori into a younger (<45 years; *n* = 9) and an older (≥45 years; *n* = 11) age group. The age cut-off of 45 years was selected based on epidemiological evidence demonstrating that the prevalence of radiographic hand osteoarthritis as osteophyte formation increases during the fifth decade of life and continues to rise progressively with advancing age [30]. Only participants in the older group were aged 50 years or older. This threshold was intended to distinguish participants with a relatively low background prevalence of age-related degenerative joint changes from those entering an age range in which osteophytes and other radiographic features of hand osteoarthritis become increasingly common in the general population. Furthermore, the selected cut-off is consistent with previous radiographic studies of rock climbers by Bollen et al. [20] where osteophytes occurred in the finger joints over 40 years of age. Thereby facilitating comparison with a climber population representing the age range in which cumulative climbing exposure and age-related degenerative skeletal changes are increasingly likely to coexist.

The average age of the young group was 33.9 years with a mean IRCRA climbing level (highest value in bouldering or lead climbing) of 27.9 and 55.8 years in the old group with a mean IRCRA climbing level of 26.5 (Table 1 and Table 2).

All participants agreed and signed an informed consent that their collected data could be used for this study. The local ethic committee gave written confirmation for our investigation.

One participant (CLIM_0028) was excluded from the analysis due to problems as the instrumented grip holds were not working properly.

No sex-based groups were defined a priori. During participant recruitment, only two contacted female climbers met the predefined performance criterion and were therefore included in the study. The two female participants were excluded from the strength analysis because sex-related differences in maximal finger flexor strength have been reported in sport climbers [35]. Given the small number of female participants, their inclusion would have introduced sex-related confounding and reduced the comparability of maximal strength measurements within this predominantly male cohort.

### 2.2. Experimental Setup and Equipment

Data acquisition using a custom-built instrumented climbing wall and an optoelectronic motion capture system was conducted at the facility of the science laboratory of the Balgrist Campus in Zurich, Switzerland. The multi-camera setup was adapted to allow for high-resolution tracking of upper limb and finger joint movements during various climbing tasks.

The marker setup, contact force, and kinematic model were the same as in our previous study [11].

#### 2.2.1. Climbing Wall

**Figure 1:**
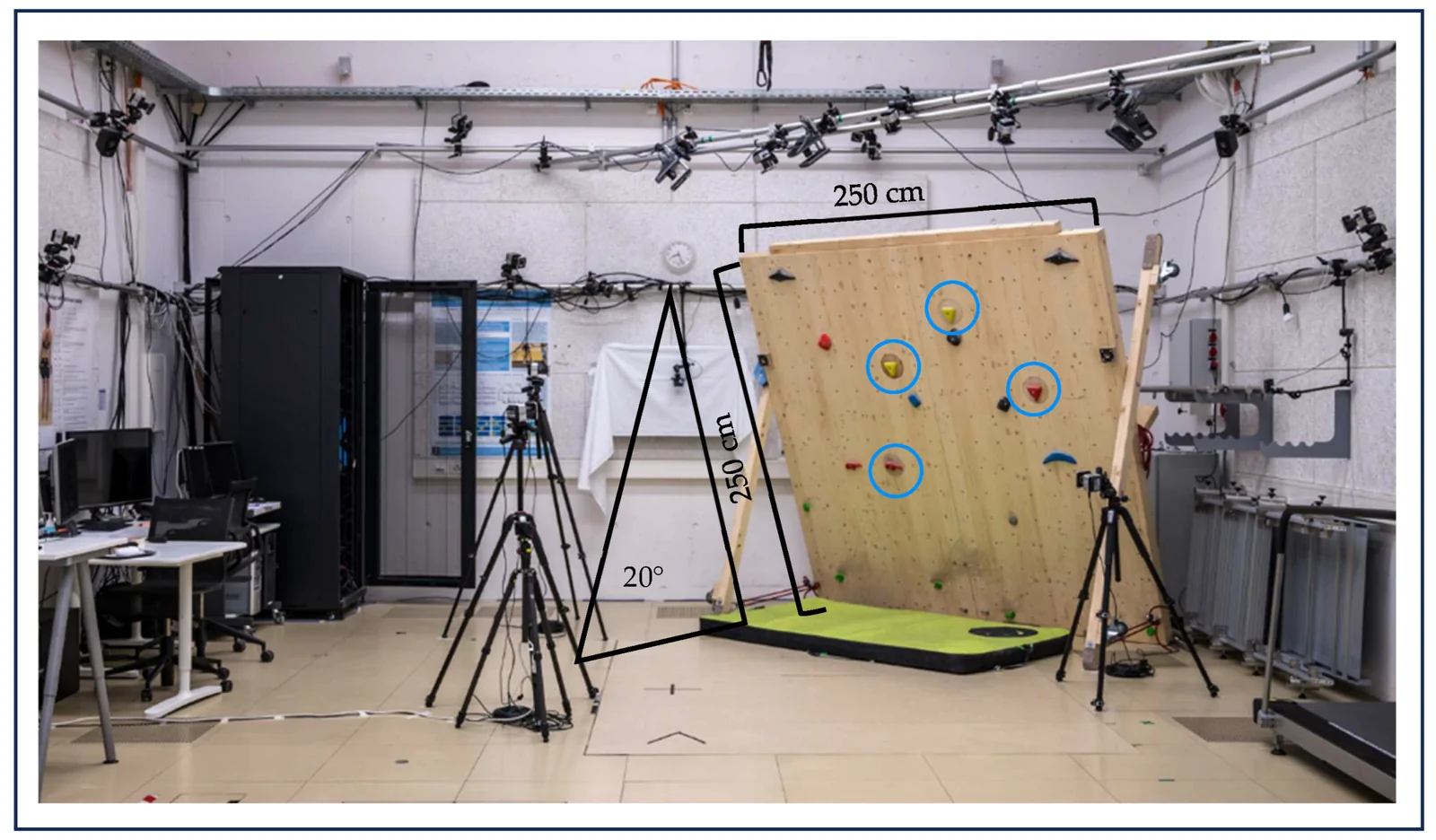
Experimental setup with the self-standing climbing wall (250 cm × 250 cm height and width, 20° angulation) and four instrumented climbing holds with custom-designed load cells (blue circles) surrounded by Motion Capture system, including nineteen infrared cameras and the computer with the VICON^®^-Nexus software.

**Figure 2:**
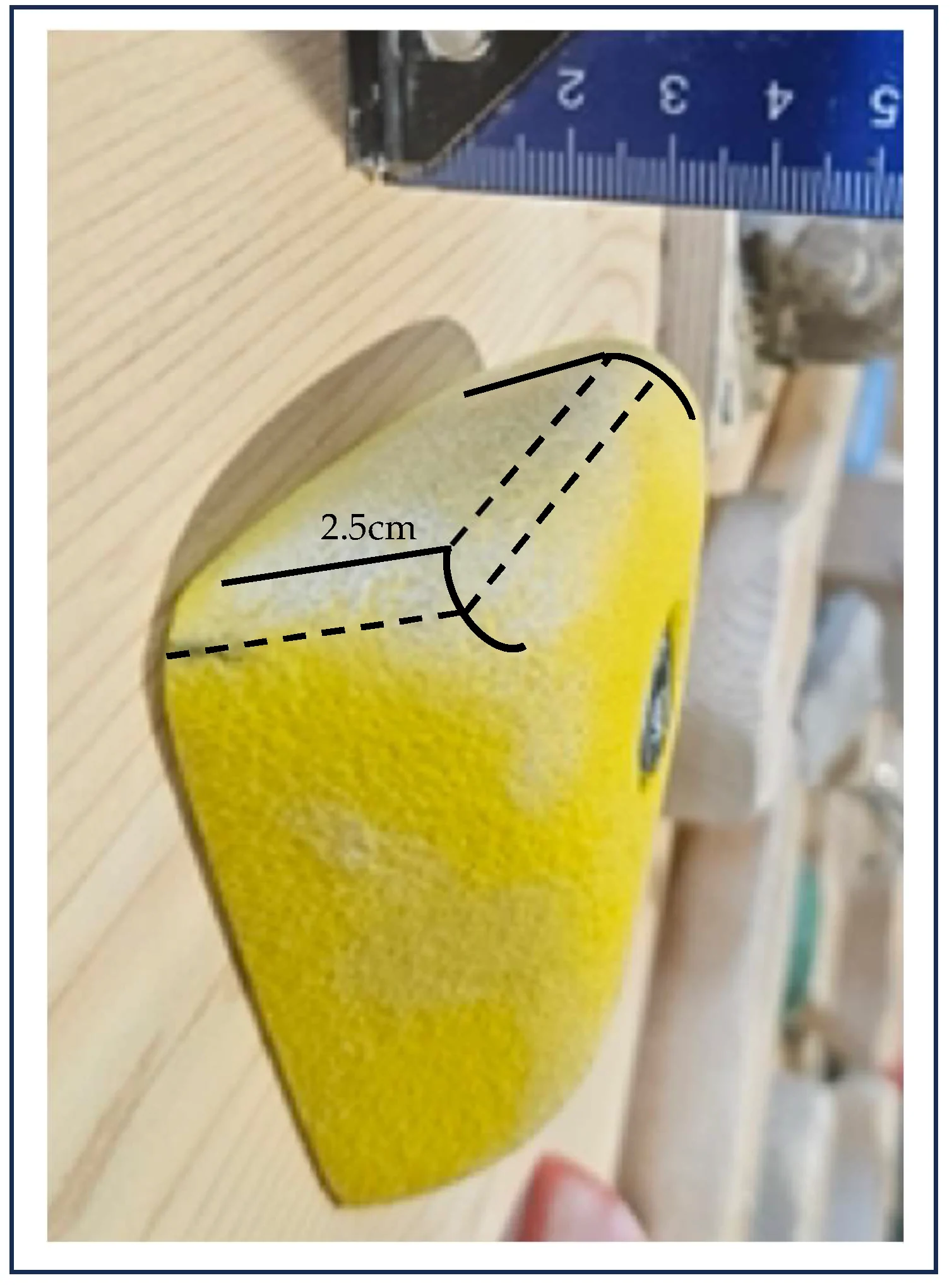
Slightly angled holding surface of 2.5 cm of the instrumented holds 3, 4 and 5 with round shaped edges. The figure illustrates the finger placement area to facilitate a better understanding of the holding surface.

A self-standing wooden climbing wall with a height and width of each 250 cm was built in a 20° angle (Figure 1). A defined boulder was set up with 6 plastic climbing holds. The holding surface was around 2.5–3.5 cm (Figure 2), horizontal to slightly angled. The distance between the holds consisted of around 60 cm to 120 cm. Six foot-holds could be used undefined.

**Figure 3:**
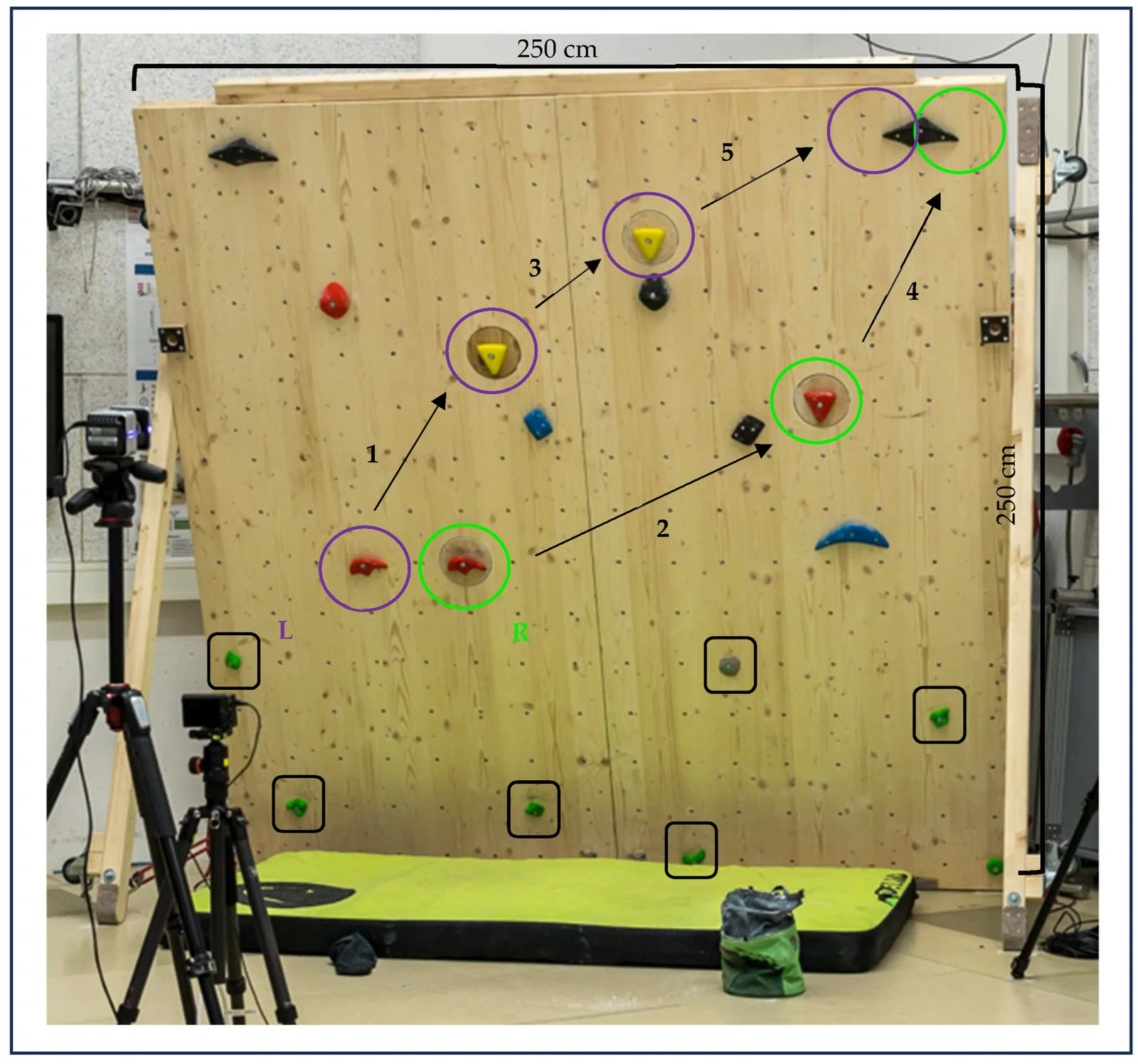
The defined climbing task consisted of five alternating hand movements, starting (1) with the left hand after the initial holds (L = left hand with purple rings and R = right hand with green rings) and ending with a matched finish hold (5). The distances between the holds was around 60 cm to 120 cm. Six foot-holds could be used undefined (black squares).

Four instrumented holds in a row measured the force during the climbing tasks, beginning with the right starting hold. No force measurement was applied on the left starting hold because the tasks started with a movement of the left hand, and with the finish hold (Figure 3).

#### 2.2.2. Force Measurement System

The four instrumented climbing holds with custom-designed load cells measured with a rate of 1000 Hz forces and moments in all spatial directions [36] and were synchronized to the optoelectronic motion capture system by a trigger signal.

#### 2.2.3. Motion Capture System

The optoelectronic motion capture system (VICON^®^ motion capture system, Oxford Metrics Ltd., Oxford, UK) included nineteen infrared cameras (16 VICON^®^ Vero v2.2, 2048 × 1088 pixels and 3 VICON^®^ MX T10, 1120 × 896 pixels) and the software VICON^®^-Nexus (version 2.3 and 2.9.2). Surrounding the set-up of the climbing wall all cameras recorded the participants in same position and adjustment (Figure 1).

### 2.3. Marker Placement

**Figure 4:**
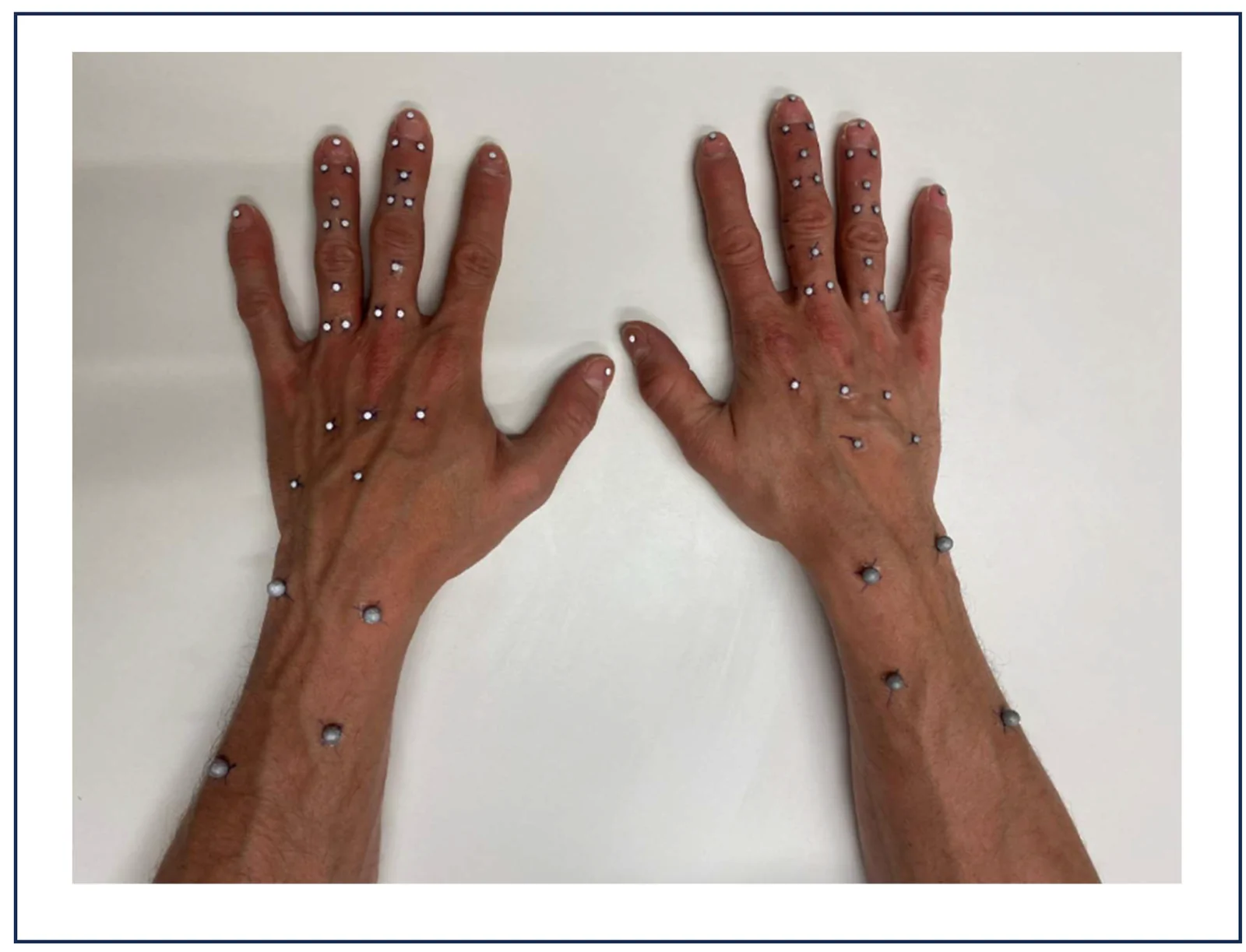
The marker set was applied, consisting of 26 hemispherical markers (3 mm diameter) on the hand and 4 spherical markers (5 mm diameter) on the forearm of each hand. Kinematic analysis was performed based on eight segments per hand, defined by the marker clusters positioned on the forearm, palm, and the proximal, intermediate, and distal phalanges of the fingers.

To track the kinematics of the fingers and the wrist, reflective markers were attached at defined positions on the dorsal side of the hand and forearm. Since pulley injuries most commonly occur in the middle and ring fingers, we only marked these two fingers to reduce tracking problems. Each phalanx of the third and fourth finger was equipped with three reflecting markers (hemispherical, 3 mm in diameter) in a triangular shape. To detect the palm, we marked the caputs of the metacarpal bones II, III and IV as well as the bases of metacarpal bones III and V with the same sized markers. The wrist and forearm were detected by spherical, 5 mm in diameter markers on the processus styloid ulnae and radius (Figure 4). Additional markers were attached to the fingertips of digits I, II und V to determine whether these fingers were actively involved in gripping the hold.

### 2.4. Tasks Instructions and Trial Protocol

#### 2.4.1. Basic Motion Task

To evaluate the active range of motions (ROM) of wrist and finger joints of each participant and calculate the center as well as axis of the joints, the climbers followed a basic motion protocol [37]. Each task was performed two times to calculate the mean values: Static neutral reference position. Full range-of-motion (ROM) of the wrist in flexion and extension. Full ROM of the wrist in radial and ulnar deviation. Full ROM of all fingers in adduction and abduction at the metacarpophalangeal (MCP) joints. Full ROM of only the middle finger in adduction and abduction at the MCP joint. Full ROM of the fingers in flexion and extension. Full ROM of only the PIP and DIP joints in flexion and extension.

#### 2.4.2. Climbing Task

The defined climbing task consisted of five movements alternating with the left and right hand. After the two starting holds, the first move was performed with the left hand, and the finish hold had to be matched with the left hand (Figure 3). The footholds were allowed to choose freely. The participants were using their own climbing shoes.

**Figure 5:**
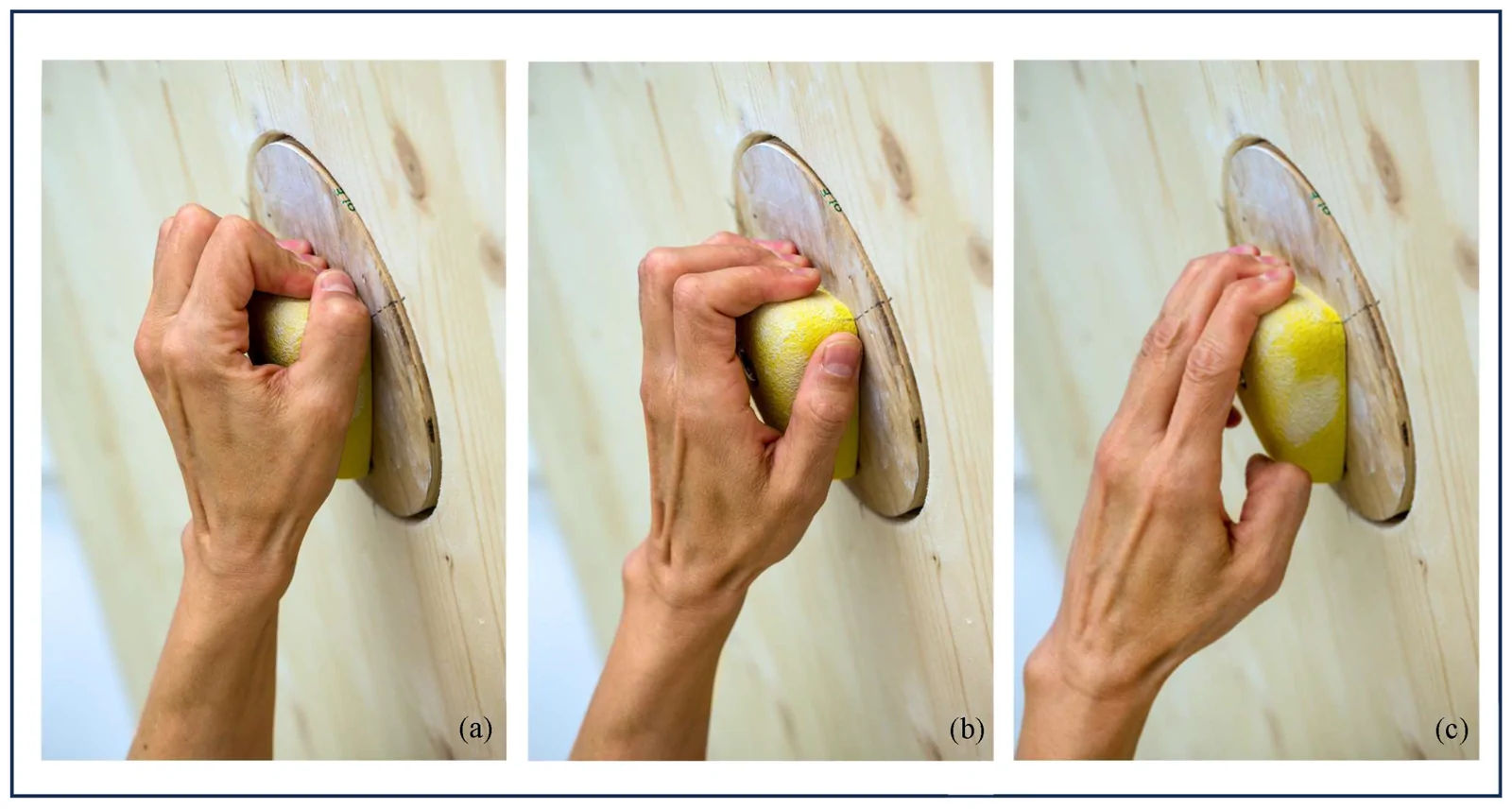
Distinct grip positions: (**a**) crimp, (**b**) half-crimp, (**c**) open-hand grip and self-selected grip (**a**–**c**).

Four climbing conditions were required: first, each climber used their personally preferred grip position. Subsequently, climbers were explicitly instructed to perform the boulder using three predefined grip position: crimp, half-crimp, and open-hand grip (Figure 5), aiming to apply each of these grip types as consistently as possible throughout the climb. The open-hand grip position was defined as climbing without using the 5th digit due to high joint angle differences in the DIP and PIP of the middle and ring finger which is described in the previous paper [11]. Three recordings were made for each condition. If the required finger position or correctly synchronized recording of the optoelectronic motion capture and force measurement systems was not achieved, or in case of marker displacement or foot contact to the bottom, the recording was repeated.

#### 2.4.3. Maximal Finger Strength Task

Additionally, a one-handed maximal finger strength test was performed using instrumented hold 4 (hold 2 for CLIM_0022) with a holding surface of 25 mm in the half-crimp and open-hand grip positions. The aim was to slowly increase the load on the hand up to the maximal achievable load. For climbers, who were able to support their whole body weight with one hand, the weight was increased by additional 10 kg attached to belt. These tasks were performed twice per task and hand, and the force as well as the kinematics were measured using the instrumented hold and the motion capture system, respectively. The same analysis was used as described in Zihlmann et al. [38].

### 2.5. Data Processing and Analysis

#### 2.5.1. Kinematic Data

The 3D reconstruction of marker positions and labeling were performed using VICON-Nexus software (version 2.3 and 2.9.2). Subsequently, the data were imported into MATLAB for further analysis. The same methodological approach as used for previous studies was used here [11,31]. Thereby, the biomechanical hand model comprised 8 rigid segments and 7 joints with 3 degrees of freedom for each hand. Segment positions and orientations were derived from marker clusters using a least-squares fit [39].

The rotations of the distal to the proximal segment were decomposed into clinically interpretable components. The flexion axes of the finger joints and wrist were determined functionally. Anatomical joint angles were calculated using the floating axis approach of Grood and Suntay [40].

The data of the right and left digits III and IV, respectively, were analyzed using the mean and standard deviation. The markers placed on the fingertips of digit I/II/V were not included in the joint angle calculations but only used to estimate whether the respective finger was in contact with the climbing hold.

#### 2.5.2. Time Normalization of Contact Forces and Kinematic Data

Phase determination in this work was conducted by calculating specific phase transition times. The underlying algorithms and threshold parameters were adapted from our previous project [11] and optimized through iterative analysis of the sensor’s force curve. Obtained phase transitions were verified against visual inspection.

(1) $$t_{0}=first\left(F_{res}>\frac{bodyweight}{35}\right)$$

$t_{0}$ denotes the grabbing phase (P1) with the first instance at which the resultant force $F_{res}$ exceeds the climber’s *bodyweight* divided by 35 (19.6 N for 70 kg) (Equation (1)). A regrip detection algorithm was implemented to adjust $t_{0}$ in cases of transient force reductions caused by hand readjustments [41].

The grabbing phase ends when the defined grip position was under full control, and the holding phase (P2) begins (*t*_1_). $t_{1}$ is determined as the first instance after an index a where the slope exceeds $\frac{bodyweight}{400}$ (slope = 1.7 N/ms for 70 kg) (Equation (2)). The parameter index a identifies the initial rise in $\frac{\partial F_{res}}{\partial t}$ exceeding $\frac{bodyweight}{180}$ (slope = 3.8 N/ms for 70 kg) (Equation (3)), ensuring that subsequent regrip events are excluded.

(2) $$t_{1}=first\left(\frac{\partial F_{res}}{\partial t}>\frac{bodyweight}{400\;ms}\right),\;t\in \left\{index\;a,\ldots ,t_{max}\right\}$$

(3) $$index\;a=first\left(\frac{\partial F_{res}}{\partial t}>\frac{bodyweight}{180\;ms}\right),\;t\in \left\{t_{0},\ldots ,t_{max}\right\}$$

Releasing phase (P3) began with the weight relief (*t*_2_), which was detected as a decline in the slope of the kinetic curve below −1.5 N/ms (Equation (4)) and ended with no grip force on the handhold (*t*_3_) detected as the last instance of $F_{res}>\frac{bodyweight}{35}$ (Equation (5)).

(4) $$t_{2}=last\left(\frac{\partial F_{res}}{\partial t}>-1.5\frac{N}{ms}\right)$$

(5) $$t_{3}=last\left(F_{res}>\frac{bodyweight}{35}\right)$$

The revised approach uses thresholds that are scaled to body weight, ensuring comparable relative thresholds across climbers. In addition, *t*_1_ is now determined solely from the current sensor data, improving accuracy and eliminating its dependence on the previous sensor. In contrast to the previous method [11], the proposed approach does not require the identification of a local minimum. This improves robustness, particularly during sideways or highly efficient climbing movements, in which a distinct local minimum may be less pronounced or absent.

For the visualization of motion patterns, the time normalization was performed separately for individual movement phases to account for differences in movement timing across subjects.

#### 2.5.3. Contact Forces During Climbing

Following the methodology described by the former published paper [11], contact forces were analyzed using MATLAB^®^ R2022b (The MathWorks Inc., Natick, MA, USA). The three force components recorded by the instrumented holds were combined to yield a resultant force and normalized to the body weight (BW) of each participant. The contact force averaged over the holding phase was compared across the different gripping conditions between young and old climbers.

#### 2.5.4. Analysis of Maximal Finger Strength

The maximal finger strength was defined as the peak force value during the one-handed finger strength task performed on the instrumented hold 4 (hold 2 for CLIM_0022), and additionally, the corresponding timepoint was determined. The joint angles at this timepoint (joint angle at maximal finger strength, JAMF) for the joints PIP3, PIP4, DIP3, and DIP4 were extracted. Due to the possibility of discontinuous joint angle curves caused by temporal invisibility of markers during the measurement, the angle of the next available frame was used in these cases.

The maximal finger strength and the JAMF were averaged across the two trials for each grip position (half-crimp and open-hand) and climber.

Furthermore, the analysis included comparisons between young and old climbers, and between the mean joint angles during maximal one-handed finger strength task (JAMF) and the mean joint angle during the holding phase of the climbing tasks (JACT) for the corresponding grip position. The mean JAMF was subtracted from JACT to determine the difference (dif) for each joint (joint: PIP3, PIP4, DIP3, and DIP4) and grip position (grip: half-crimp and open-hand, Equation (6)).

(6) $$Dif_{joint_{grip}}={JAMF}_{joint_{grip}}-JACT_{joint_{grip}}$$

### 2.6. Detecting Osteophytes of the DIP and PIP Joints via Ultrasound Examination

Ultrasound examination was performed using the Samsung ultrasound device (Medison HM70A, 15 MHz hockey stick probe LS 6, linear view) to detect osteophytes on the dorsal side of the DIP joints, limiting hyperextension, as well as on the palmar side of the PIP joints, limiting flexion, of the 3rd and 4th phalanges of all participants. Osteophyte formation was assessed at the DIP joints and categorized as absent (−; no osteophytes) or pronounced (+; osteophytes >1 mm). At the PIP joints, osteophytes were additionally classified as mild (+; 0.5–1 mm) or severe (++; osteophytes >1 mm on both sides of the joint).

The examination was obtained in full extension of the fingers in a longitudinal imaging plane. The examination of all climbers was performed by the same experienced hand surgeon.

### 2.7. Statistics

#### 2.7.1. Kinematics During Climbing

The joint angles during the holding phase for each of the different grip conditions (self-selected, crimp, half-crimp, open-hand) was compared between young and old climbers. Normality and homogeneity of variances of each metric of interest were first assessed using the Shapiro–Wilk test and Bartlett’s test. Based on these results, either an independent *t*-test or a Wilcoxon rank sum test was applied for group comparison (alpha 0.05). We hypothesized that older climbers would adopt a more open grip configuration due to our previous study.

#### 2.7.2. Self-Selected Grip Position

The self-selected grip position was compared with the predefined grip position: the crimp, half-crimp and open-hand grip position. Mean differences between the self-selected grip and each predefined grip position were calculated to determine which it most closely resembled.

#### 2.7.3. Analysis of Finger Force

The data of the contact force during climbing and of the maximal finger strength task were tested for normal distribution and equal variance using the Shapiro–Wilk test and Bartlett’s test and subsequently the corresponding statistical test was applied (Wilcoxon rank sum test, independent *t*-test).

Invalid trials were repeated. Incomplete data were identified, and climbers were excluded for the corresponding analysis. For the maximal finger strength task, 10 old and 7 young climbers were compared. The joint angles of 9 old and 7 young climbers were included in the comparison after excluding climbers with incomplete data (Table 1).

#### 2.7.4. Association Between Osteophytes and Joint Angles During Climbing

Spearman’s rank correlations were calculated between the development of dorsal osteophytes at the DIP joint and the corresponding mean DIP angle as well as between palmar osteophytes at the PIP and the mean PIP flexion angles during the holding phase of the climbing task. The analyses were conducted separately for each of the four grip conditions.

## 3. Results

The participants analyzed in this study consisted of two age groups of experienced climbers (Table 2). The young group, under 45 years, had an average age of 33.9 years (SD 6.34), where the counterpart, over 45 years, had a mean age of 55.8 years (SD 4.74). The mean climbing performance level (highest IRCRA value in bouldering or lead climbing) was 27.9 IRCRA for the young and 26.5 IRCRA for the older climbers without significant difference (*p* = 0.11). The training hours per week and height had no significant differences between the age groups. The BMI of the young group was 22.8 (SD 1.47) and of the old group 22.86 kg/m2 (SD 2.49) (*p* = 0.95) without any significant differences. The mean arm span consisted of 181.7 cm (SD 7.1, *p* = 0.87).

### 3.1. Reduced PIP Flexion and DIP Extension of Older Climbers During Holding Phase in Crimp, Half-Crimp and Self-Selected Grip Position

**Figure 6:**
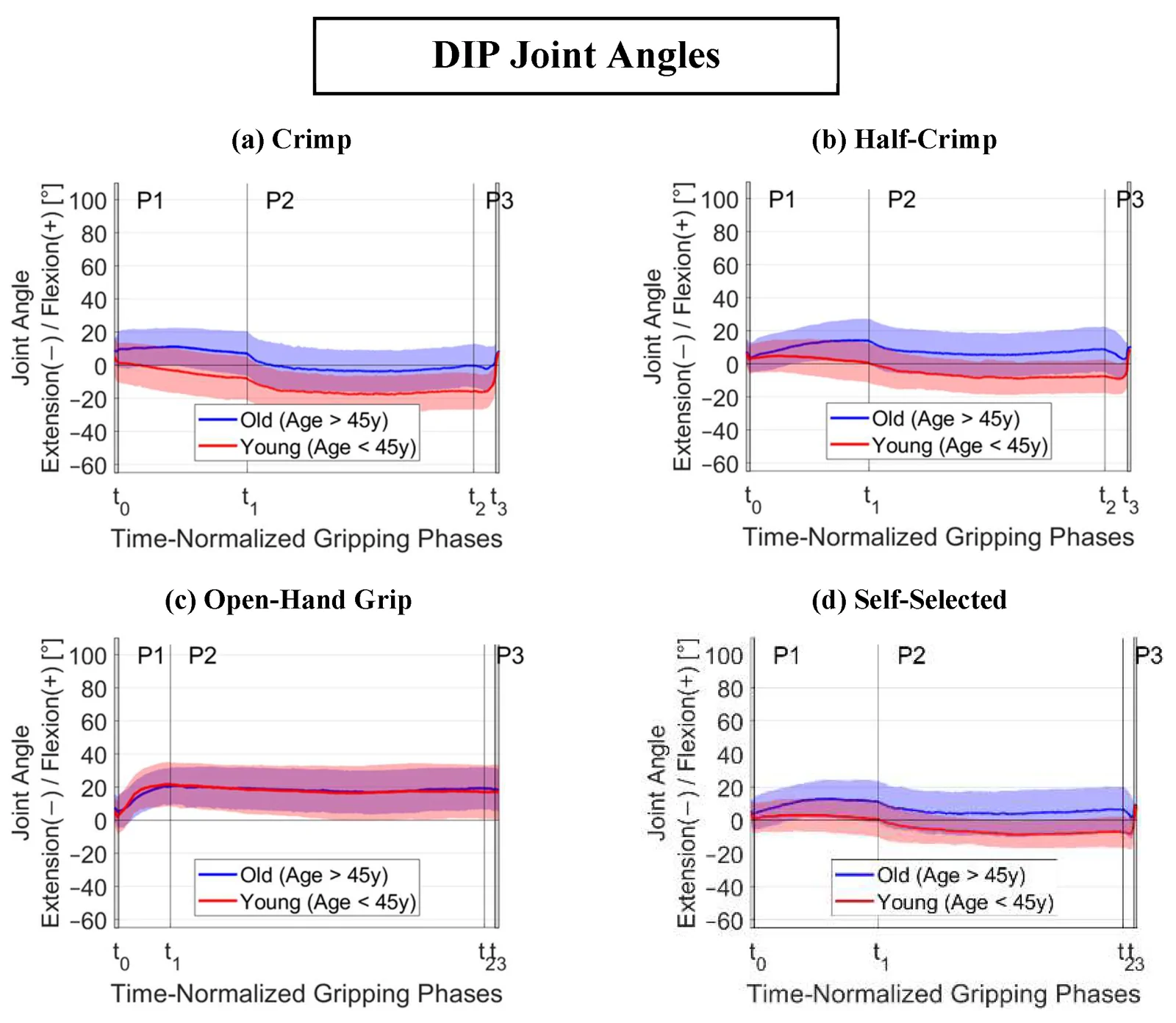
Mean (solid line) and standard deviation (marked area) of left and right DIP III and IV extension-flexion angles (in ° degrees) in crimp (**a**), half-crimp (**b**), open-hand (**c**), and self-selected (**d**) grip positions for younger (red; *n* = 9) and older climbers (blue; *n* = 11) during phase-based time-normalized gripping phases (P1 = gripping, P2 = holding, P3 = releasing).

During the holding phase (P2) of the tested grip positions, significant age-related differences were observed in finger joint angles. At the DIP, marked group-specific differences were evident. Younger climbers showed a DIP-hyperextension (negative values) in the crimp and half-crimp positions, while older climbers exhibited less hyperextension in the crimp position and slight flexion in the half-crimp position. These differences were highly significant (*p* < 0.0001). The self-selected grip position, corresponding to the half-crimp position, showed similar trends. The mean differences in DIP joint angle for the crimp, half-crimp and open-hand grip to the self-selected grip position were 8.0° (SD 3.6), −1.2° (SD 3.9) and −18.7° (SD 9.7). No significant group difference was found in the open-hand grip position (*p* = 0.83) (Table 3, Figure 6).

**Figure 7:**
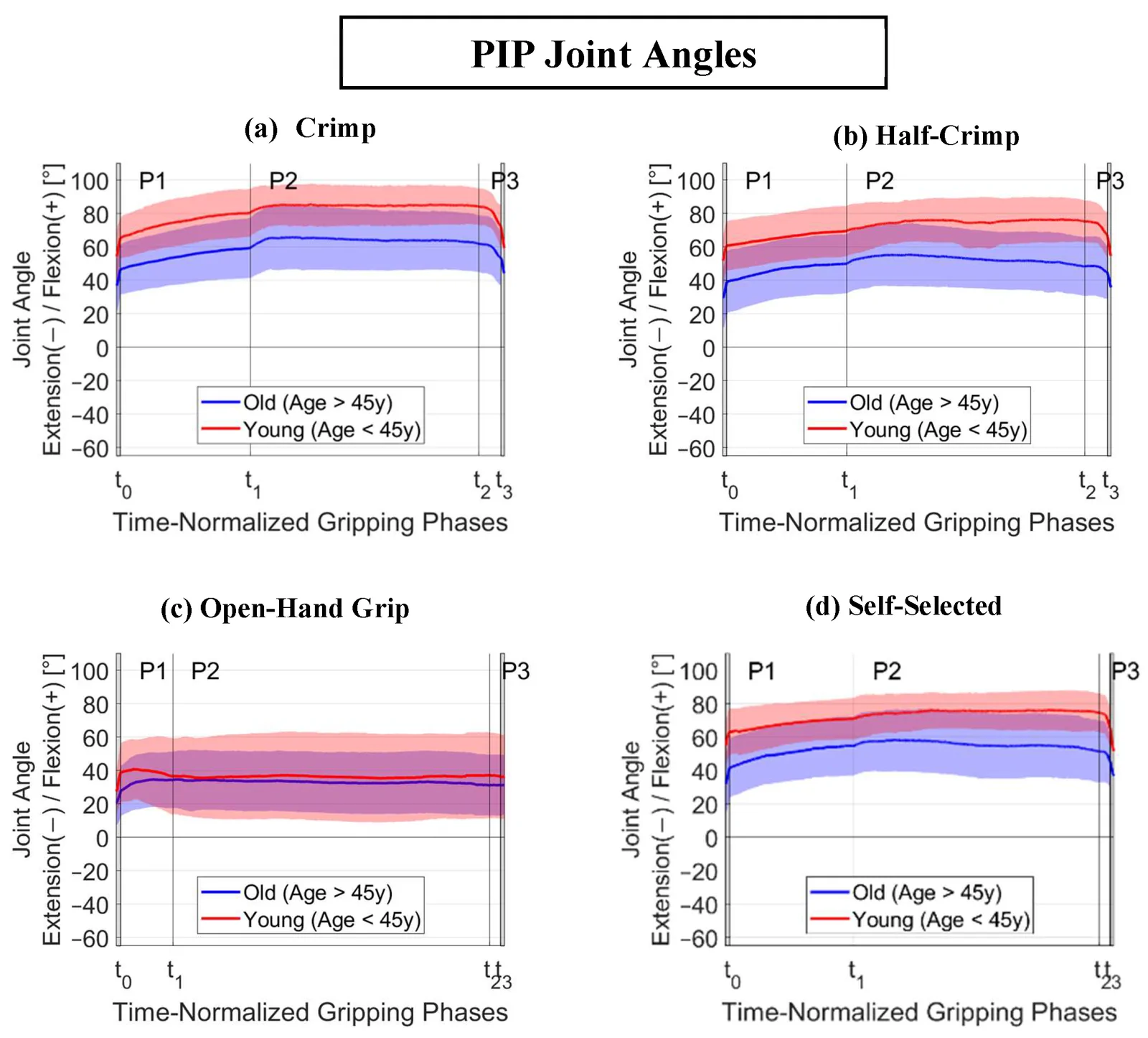
Mean (solid line) and standard deviation (marked area) of left and right PIP III and IV extension-flexion angles (in ° degrees) in crimp (**a**), half-crimp (**b**), open-hand (**c**), and self-selected (**d**) grip positions for younger (red; *n* = 9) and older climbers (blue; *n* = 11) during phase-based time-normalized gripping phases (P1 = gripping, P2 = holding, P3 = releasing).

In the crimp and half-crimp grip position, older climbers exhibited substantially reduced PIP flexion compared with younger climbers (Table 3, Figure 7), both with highly significant differences (*p* < 0.0001). In the self-selected grip position, which biomechanically corresponded closely to the half-crimp position, a similar pattern was found in both groups. The mean differences in PIP joint angle for the crimp, half-crimp and open-hand grip to the self-selected grip position were −9.4° (SD 5.4), 2.0° (SD 5.0) and 30.0° (SD 14.1).

By contrast, in the open-hand grip position, no significant difference was observed between the age groups (*p* = 0.44) (Table 3, Figure 7).

Overall, in the crimp and half-crimp grip positions, older climbers had less PIP flexion and a neutral to slightly flexed DIP posture, whereas younger climbers demonstrated greater PIP flexion combined with DIP hyperextension.

Regarding active ROM, a significantly reduced PIP flexion and wrist extension capacity of older climbers was observed. For the other joints, no significant group effects were found (Table 4).

The mean PIP flexion of the active ROM during basic motion task was 24.1° (SD 9.3, range 4.3–61.3) greater than the flexion during the crimp position, so that all climbers did not use their full possible PIP flexion. The mean DIP extension of the active ROM during the basic motion task was 3.2° (SD 12.7, range −27.6–31.4) and in 26 out of 80 DIP joints the maximum extension was exceeded during the crimp position due to passive extension. During the open-hand grip position, the full DIP flexion was not used as the mean DIP flexion during motion task was 45.1° (SD 16.5, range 10.7–78.5).

### 3.2. Self-Selected Grip Position Corresponded to the Half-Crimp

The self-selected grip position at the PIP in most climbers (*n* = 12; 4 young, 8 old) lies between their individual half-crimp and crimp position. Still, 6 climbers (3 old, 3 young) spontaneously used a grip position that was slightly more open than their individual half-crimp position. In two cases (2 young), it exactly matched their half-crimp position. The mean absolute difference between the individual self-selected grip position and the half-crimp was 4.3° (SD 3.3°), whereas larger differences were found compared to the crimp (mean 9.4°, SD 5.4) and the open-hand grip position (30.0°, SD 14.1°).

### 3.3. Grip Force During Climbing

Young climbers applied greater forces to the instrumented handholds during the holding phase across all grip conditions (Table 5) compared to older climbers, whereas forces exerted through the feet were not quantified.

### 3.4. Negative Correlation of Osteophytes with PIP Flexion and Positive Correlation with DIP Flexion

In young climbers, osteophytes could be sonographically verified in 3 PIP (8.33%) and 6 DIP (16.67%) joints of 36 joints whereas in older climbers, osteophytes could be seen in 30 PIP (68.18%) and 36 DIP (81.82%) joints of 44 joints.

Two young and four old climbers had osteophytes only on the dorsal side of the PIP joints.

**Figure 8:**
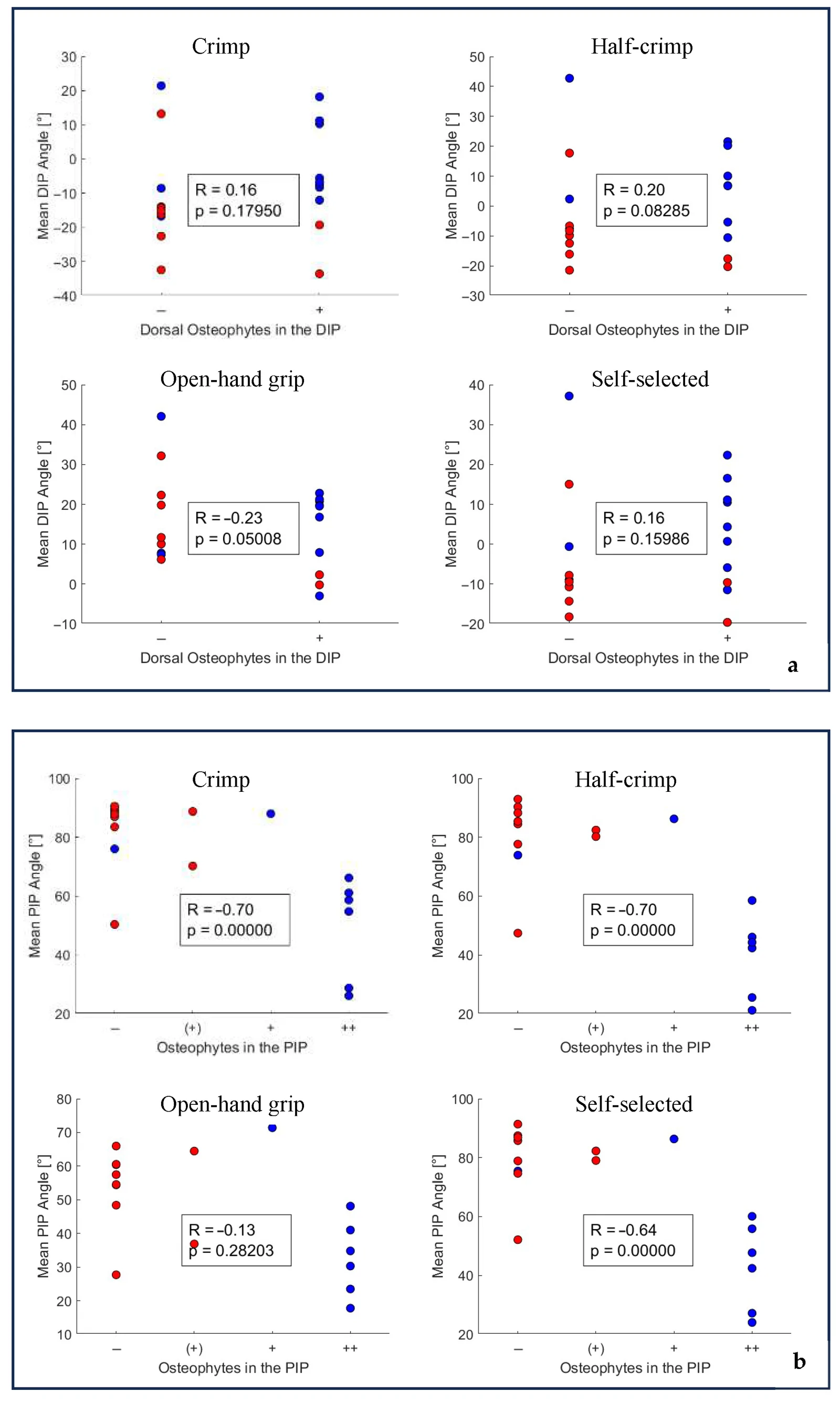
Spearman correlation (R) between mean DIP (**a**) and PIP (**b**) angle (in °) to absent (−), mild ((+)), pronounced (+) and severe (++) osteophytes in the PIP and DIP of digits III and IV (blue dots: old climbers (*n* = 11); red dots young climbers (*n* = 9)). *p*-value < 0.05 denote significant differences.

The distal interphalangeal (DIP) joint showed a trend for reduced hyperextension with the presence of osteophytes during the crimp (*p* = 0.18, R = 0.16) and half-crimp grip (*p* = 0.08, R = 0.20) as well a less DIP flexion during the open-hand grip (*p* = 0.05, R = −0.23), with a weak correlation (Figure 8a) The development of osteophytes in the PIP joints of climbers is significantly (*p* < 0.00001) associated with reduced proximal interphalangeal (PIP) joint flexion during crimp (R = −0.70), half-crimp (R = −0.70) and self-selected grip positions (R = −0.64) (Figure 8b).

### 3.5. Negative Correlation Between DIP and MCP Joint Compared to Maximum PIP Joint Flexion

**Figure 9:**
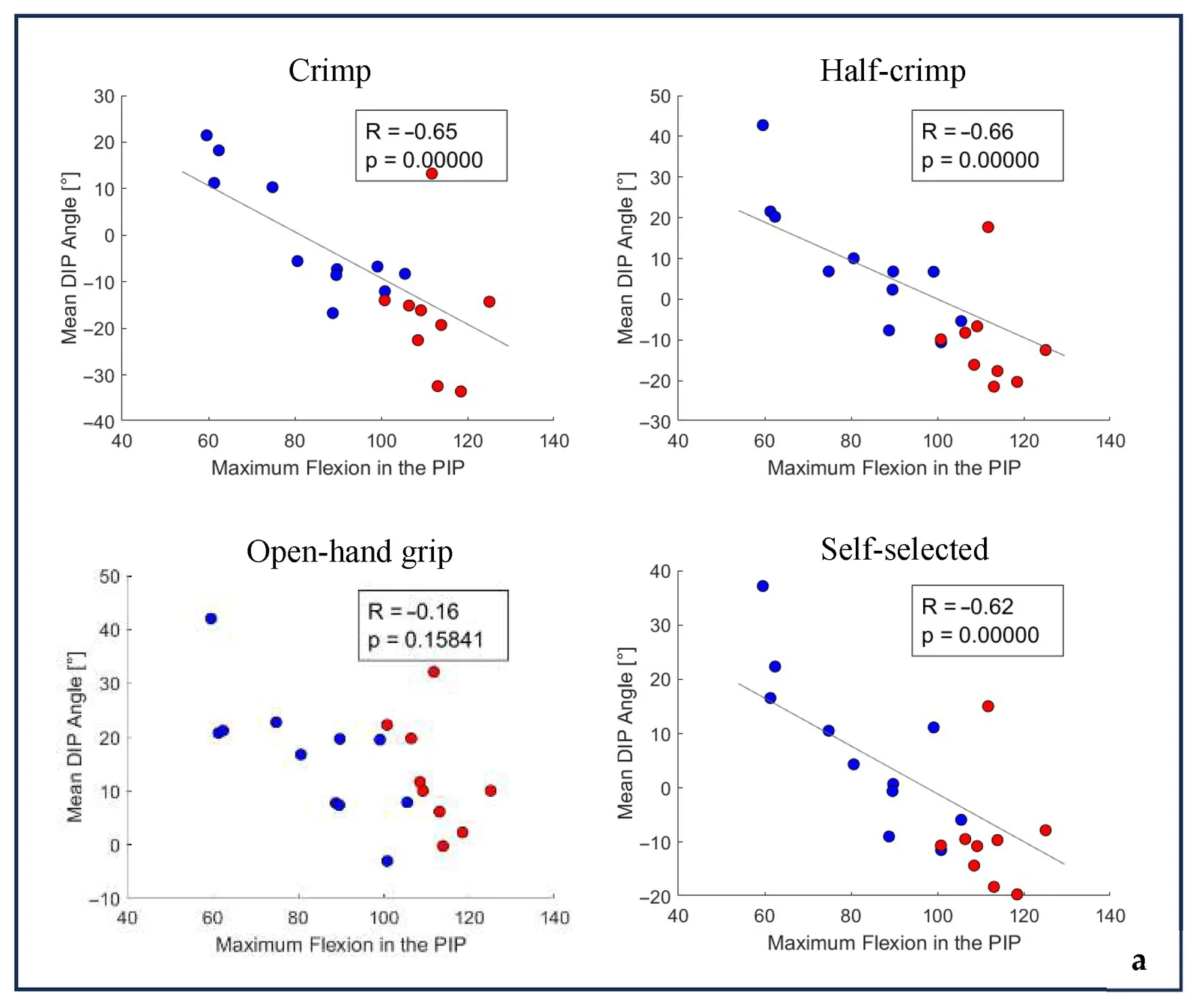

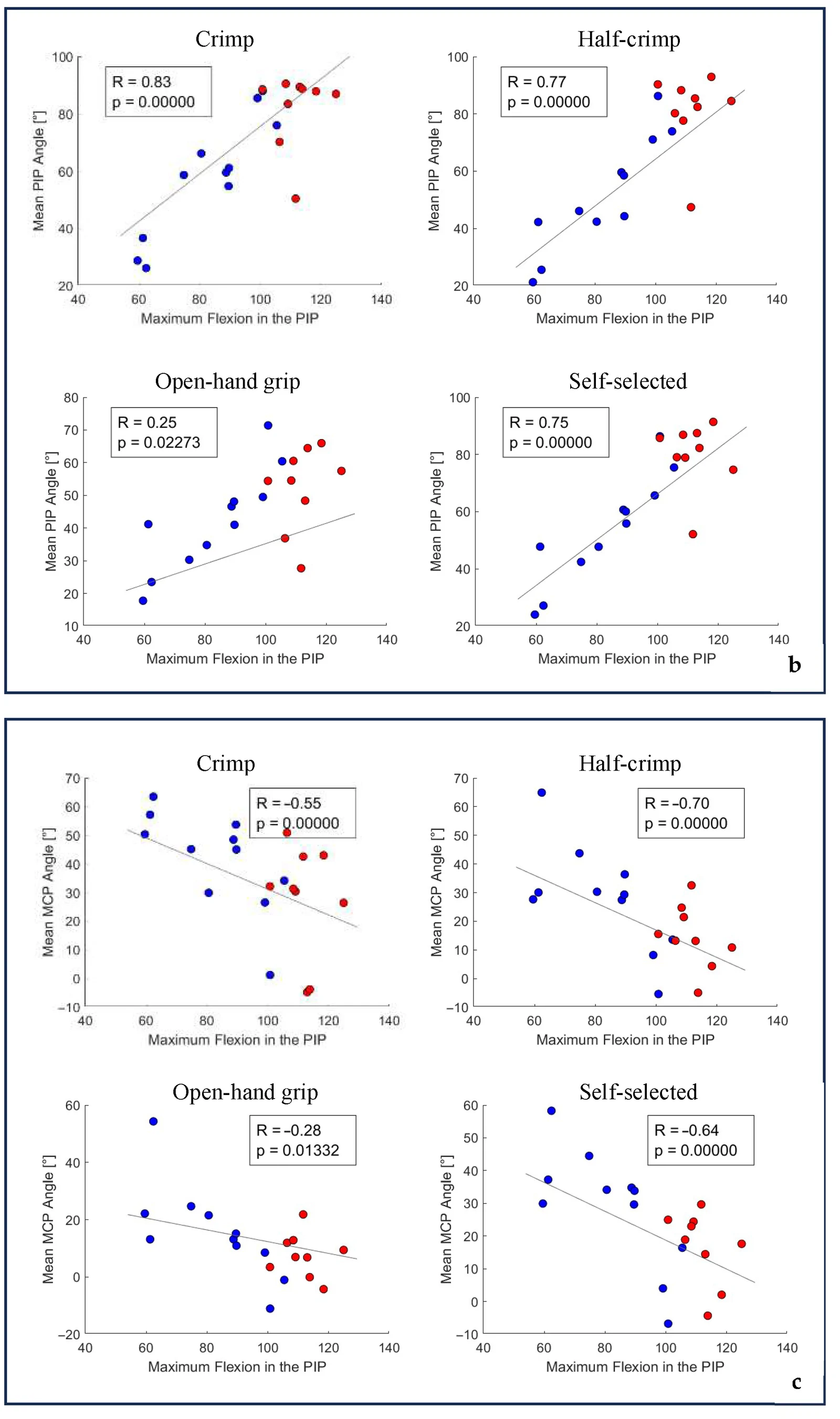
Spearman correlation (R) between mean DIP (**a**), PIP (**b**) and MCP (**c**) angle (in °) of digit III and IV during crimp, half-crimp, open-hand grip and self-selected grip position to maximum flexion in the PIP joint (in °) during basic motion task (blue dots: old climbers (*n* = 11); red dots young climbers (*n* = 9)). *p*-value < 0.05 denote significant differences.

Joint angles recorded during climbing in the crimp, half-crimp and self-selected grip positions exhibited a significant negative correlation for the DIP (Figure 9a) and MCP joint (Figure 9c) when compared with the maximum achievable PIP flexion during the basic motion task as well as a positive correlation for the PIP joint (Figure 9b). So that old climbers with less flexible PIP joint during the basic motion task had relatively more flexion in the DIP and MCP joints during climbing. Furthermore, lower maximal PIP flexion during the basic motion task was generally associated with reduced PIP flexion during the climbing tasks.

### 3.6. Higher Maximal Finger Strength in Half-Crimp Grip Compared to Open-Hand Grip Position Without a Difference Between the Age Groups

**Figure 10:**
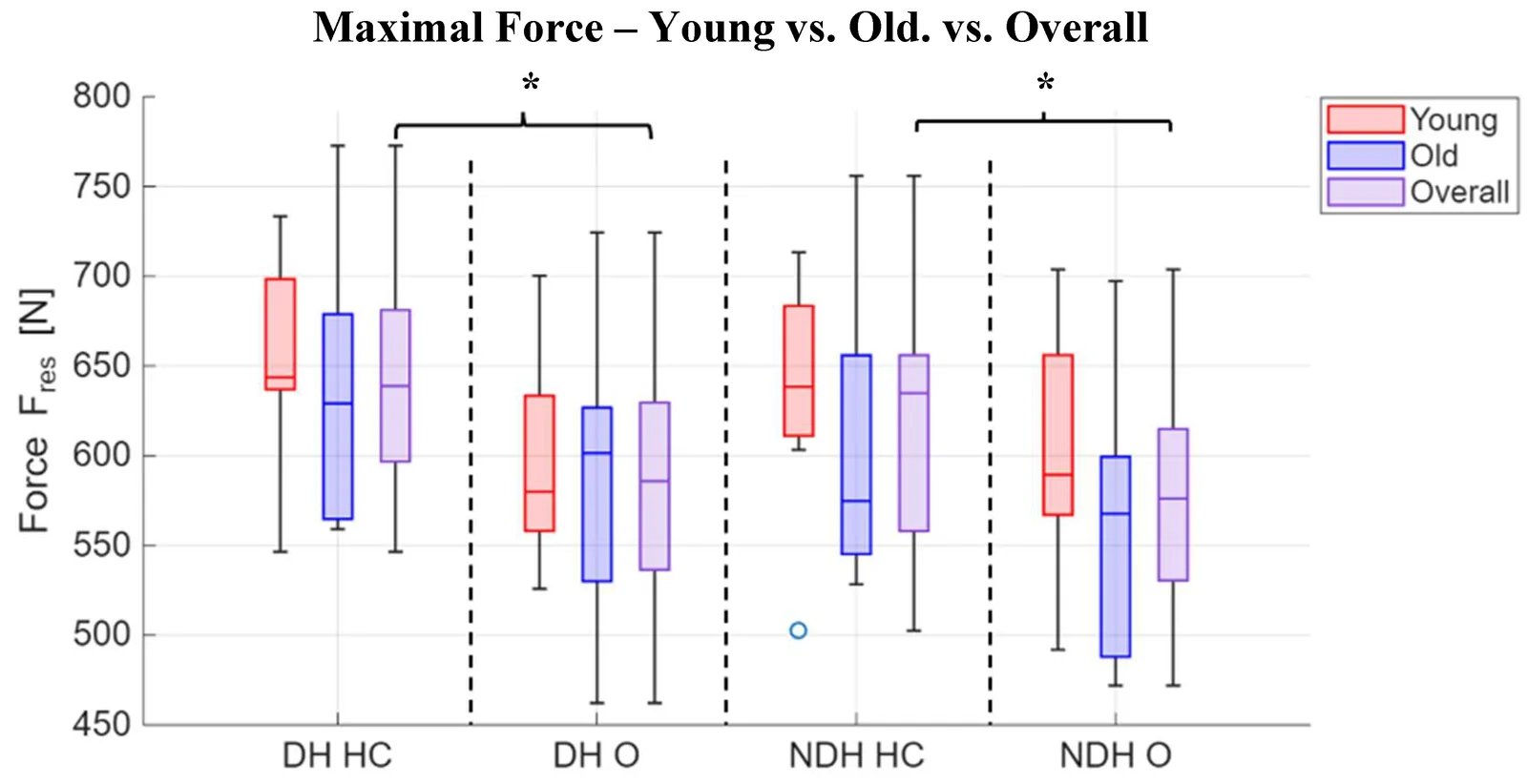
Maximal finger strength (in N) of young (*n* = 7) and old (*n* = 10) male climbers and overall (*n* = 17) with grip positions half-crimp (HC) and open-hand (O) conducted with their dominant (DH) and non-dominant hand (NDH) (* show significant differences).

The analysis of maximal strength included only male participants due to the limited comparability of sex-specific maximal finger strength. The maximal finger strength in the half-crimp position is significantly higher compared to the open position over all climbers (*p* < 0.05) (Figure 10) with mean maximal values of 628.7 N (SD 67.4 N) for the half-crimp position and 584.1 N (SD 69.0 N) for the open-hand grip position. The old climbers achieved mean maximal values of 618.6 N (SD 70.1 N) in the half-crimp position and 572.8 N (SD 72.6 N) in the open-hand grip position, whereas the young climbers achieved mean maximal values of 643.0 N (SD 63.1 N) for the half-crimp position and 600.2 N (SD 62.3 N) for the open-hand grip position. No significant differences were found between the performance of the dominant and non-dominant hand for both young and old climbers. Also, no statistically significant differences were found for the maximal finger strength between young and old climbers for the half-crimp or open-hand grip position with the dominant or non-dominant hand.

### 3.7. More Open Finger Position in the Maximal Measured Force Task Compared to the Climbing Task

Regarding the JAMF, the DIP angles were significantly different from the JACT for the half-crimp and open-hand grip position with both hands (*p* < 0.0001), whereas the PIP joint angles were significantly different for the open-hand grip position with both hands (*p* < 0.05) but not for the half-crimp grip position (DH: *p* = 0.50 and NDH: *p* = 0.51).

Negative PIP angular differences showed a lower flexion in the PIP joints as well as less hyperextension in the DIP joints and therefore a more open position for both grip positions compared to the climbing task. The joint angle differences between JAMF and JACT showed the highest deviations at DIP3 and DIP4 joints compared to the other joints evaluated (wrist, MCP3 and 4, and PIP3 and 4). The DIP3 and DIP4 joint angle differences in both hands and tasks showed positive differences (Table 6), indicating that the DIP was less hyperextended and more flexed. For the PIP joint angles, the mean differences were negative, so that the PIP was less flexed, except for the PIP3 with the half-crimp grip position with angular deviations of 0.2° (SD 9.0°) and 0.7° (SD 11.1°) for the dominant and non-dominant hand, respectively (Table 6). No statistically significant age-group differences in the PIP or DIP angular differences were observed.

## 4. Discussion

### 4.1. Older Climbers Have a Reduced PIP Flexion and DIP Hyperextension During Climbing

This study demonstrates that older climbers had a markedly less flexed PIP and less extended DIP joint during the crimp, half-crimp and self-selected grip position. In addition, older male climbers applied lower forces to the handholds across the different grip conditions (old: 40.5% BW, young: 44.4%).

As shown in our previous publication [19], the DIP joint exhibited the greatest hyperextension in the crimp position (~−15.7° in young and ~−2.2° in old climbers). The half-crimp position showed less hyperextension to slight flexion (~−6.7° in young and ~7.2° in old climbers), whereas the open-hand grip position resulted in the greatest DIP flexion (~18.5° in young and ~18.4° in old climbers).

In contrast, the PIP joint showed the highest mean flexion in the crimp grip (~85° in young and ~65° in old climbers), intermediate flexion in the half-crimp grip (~75° in young and ~53° in old climbers), and the least flexion in the open-hand grip position (~37° in young and ~33° in old climbers).

Nevertheless, similarly to our previous publication [19], we did not observe the expected PIP flexion of 90–100° during the crimp grip or 0–10° during the open-hand grip position, nor the expected DIP flexion of 50–70° during the open-hand grip that has been reported in other studies [14,41,42], when quantifying finger joint angles directly during climbing activities. Marker visibility, particularly at the distal interphalangeal (DIP) and proximal interphalangeal (PIP) joints during the crimp position, continued to pose a challenge but improved clearly to our previous study, due to multiple cameras and higher-resolution devices [19].

The maximal DIP flexion (old: 62.2°, young: 65.2°) measured during maximal finger flexion-extension trials did not differ between the age groups, and 9 DIP joints (8 old, 1 young) were unable to attain the expected 50° of flexion. In contrast, maximal PIP flexion observed during the basic motion tasks was significantly reduced in older climbers (86.6°) compared to young climbers (112.3°), whereby 25 out of 44 joints in the older group did not achieve the expected PIP flexion angle of 90°, whereas all young climbers exceeded >90°.

Hence, some climbers did not have the DIP and/or PIP flexion capacity to attain the expected crimp or open-hand grip positions. However, it was also observed that maximal flexion exceeded the flexion angles achieved during climbing. Concluding, that all climbers did not use their full range of motion during climbing. This may be attributed to the effects of finger loading, the elasticity of the finger tendons and ligaments, opposing forces generated during climbing, as well as passive opening torque acting on the joints.

Additionally, in our study we observed a negative correlation between climbers with a reduced PIP joint flexion and an increased MCP and DIP joint flexion, which may reflect a compensatory mechanism for limited PIP flexion. Further research should be conducted.

#### Osteophyte-Associated Alterations in Finger Joint Kinematics Without Impairment of Maximal Finger Strength

In this study, older climbers over 45 years had a higher prevalence of base osteophytes in both the PIP and DIP joints compared to the young climbers. The development of osteophytes and related degenerative changes might attribute to sustained high mechanical loads during specific grip positions, particularly the crimp grip, which imposes substantial stress on these joints over years of climbing activity, which has been described in other previous studies [18,21,23,32,33]. Extreme finger positions, such as the crimp position, might leading to increased joint stress, reduced articular congruency, and focal high-pressure loading as documented in other joints (e.g., hip, shoulder, knee) [13,14,15,16]. Bollen et al. reported radiologically evident osteoarthritic changes in the finger joints of climbers from the age of 40 [20] and hypertrophic bone responses have been detected even after 10 years of follow-up [24]. Longitudinal imaging of high-level climbers revealed progressive thickening of the flexor tendons and pulleys over a decade, indicating that repetitive high-load climbing induces chronic soft-tissue remodeling that may also limit joint angulation [19]. Increased cartilage and osteophyte changes in the PIP and DIP joints have been reported most frequently in digits III and IV [22,24,25] and were a focus in this study. Notably, this pattern differs from typical genetically driven hand osteoarthritis [26,27].

Osteophyte formation, particularly the pathognomonic palmar neck osteophytes [42], is associated with reduced joint angulation with reduced PIP flexion and DIP hyperextension. In our study, older climbers had significantly less PIP flexion capacity (86.6°) compared to the young climbers (112.3°). Consequently, older climbers tended to have a more open grip position, as reflected by significant negative correlations between age and PIP angulation during the crimp grip (Table A1, R = −0.54, *p* = 0.014), as well as between age and PIP range of motion (Table A2, R = −0.526, *p* = 0.017). The observed associations between the higher prevalence of osteophytes in older climbers and reduced PIP and DIP joint angulation cannot be interpreted as causal due to the cross-sectional design. Nevertheless, they may indicate that older climbers with a higher prevalence of osteophytes tend to use a more open and joint-protective grip position. Additionally, osteophyte limitation due to bony impingement during climbing is unlikely as the results of the basic motion task showed greater PIP and DIP flexion. In accordance, older climbers applied lower finger forces, presumably due to more reliance on foot support or a less dynamic climbing style, which further reduces mechanical stress on the finger joints. The underlying factors cannot be determined based on this study. Potential explanations include cumulative mechanical loading associated with long-term climbing exposure as well as preserved joint adaptations. Further research is needed to clarify these mechanisms.

A more open grip position may suggest that older climbers generate less grip force during the crimp or half-crimp grip. However, our findings demonstrated no differences in maximal finger strength between the age groups, suggesting that osteophyte-associated reductions in PIP and DIP joint angulation do not impair maximal finger strength. This climbing pattern may help reduce degenerative joint changes for younger climbers without compromising performance.

Furthermore, these adaptations may even have a protective effect on the flexor tendon-pulley system under high loads by reducing joint angulation as the crimp grip is associated with more pulley injuries [2].

Consistent with this, pulley injuries are rarely observed among older climbers in our clinical practice. Pastor et al. [24] also demonstrated that climbers with degenerative joint changes could generate high maximal finger forces. The reason may result from adaptations in muscle force of the flexor digitorum superficialis which generates more force in an open-hand grip position [12].

### 4.2. Open-Hand Grip

In our former publication, Fischer et al. (2024), inter-individual differences in PIP joint flexion during the open-hand grip position were observed, which was assumed to be personal habit for use or non-use of the little finger [11]. In that study, mean PIP joint flexion was higher (left PIP3: 58.6°, right PIP3: 49.4, left PIP4: 31.7°, right PIP4: 17.9°) compared to this current study. The relatively short length of the little finger likely contributes to increased PIP joint flexion in the other fingers, especially the middle finger, due to finger length proportions.

In this study, participants were instructed not to use their little fingers during the open hand grip and we observed that the average measured flexion angle of the PIP joints was 33° (SD = 17.1°, range 4.1–71.4°) among older climbers and 37° (SD = 24.4°, range −3.9–74.1°) among younger climbers and of the DIP joints was 18.4° (SD = 11.5°, range −5.9–42.1) among older and 18.5° (SD = 14.4°, range −10.9–38.2) among younger climbers. In the previous described paper [43], the open-hand grip assumed a PIP flexion of 0–10° and a DIP flexion 50–70°. So, in our study we saw in an open hand grip position without using the little finger a slightly less flexed PIP joint and more flexed DIP joint as well as less interindividual differences than in our previous study, taking into account that the climbing holds and route setting differed.

From a mechanical perspective, the open-hand grip with extended PIP joints significantly lowers the bowstringing effect of flexor tendons and thus reduces strain on the A2 and A4 pulleys, even though body weight is supported by fewer fingers (excluding the little finger). This redistribution of forces reduces the risk of stress-induced pulley injuries, which align with clinical observations that climbing with an open-hand grip remains a safer strategy after pulley rupture than climbing with crimp or half-crimp grips in which PIP flexion is pronounced and tendon loads are maximal [9].

Moreover, previous studies have suggested that the open-hand grip provides a biomechanically favorable joint position that balances force generation with reduced joint strain, making it an essential technique for injury prevention and rehabilitation in climbing athletes (Bacon et al., 2017; Gilmore et al., 2024) [44,45]. Avoiding full finger flexion, particularly at the PIP joints, mitigates the excessive compressive forces associated with the use of crimp positions, which have been linked to a higher incidence of finger pulley injuries (Schweizer et al., 2001; Morrison et al., 2007) [43,46].

Nevertheless, in this study a few climbers demonstrated a PIP4 flexion over 40° in the open-hand grip position without using digit 5. Two participants (CLIM_40 and CLIM_29) showed this pattern bilaterally, one climber (CLIM_22) only on the left hand, and two climbers (CLIM_23 and CLIM_25) only on the right hand. All of these climbers, with the exception of CLIM_40, belonged to the younger age group. This pattern may be attributable to individual grip preferences and the unusual strategy of not using the little finger. In the video analysis, these climbers frequently displayed a flexion of the little finger prior to contact to the hold, which was observed with increased flexion of the remaining fingers. So, the open-hand grip should also be considered critical during pulley injuries and should be carefully assessed along with how the individual climber actually executes the open-hand grip.

### 4.3. Self-Selected Grip Position Corresponded to a Half Crimp Position

The self-selected grip position varied among participants but was generally less flexed (~76° of the young climbers and ~56° of the old climbers) than the crimp and corresponded to the climber individual half-crimp position. These data show that, even when completing a relatively high-grade boulder problem, the climbers intuitively adopted a half-crimp position, although maximal force can be generated in the crimp position [43]. The boulder might have been too easy for the climbers and thus the crimp position was unnecessary. Since the holding surface of the climbing hold measured 25–35 mm with a round shaped edge (Figure 2), they might have been too large for the climbers, whose mean distal phalanx length was approximately 30 mm.

Although the grip positions were standardized across all participants to maintain experimental control, each climber naturally exhibits individual climbing preferences. These preferences influence how frequently and intensively different grip positions are used during habitual climbing. As a result, climbers may have varying strength levels and conditioning in these grip positions, which could affect the biomechanical outcomes observed in this study.

### 4.4. A More Open Hand Grip Position Was Observed During Maximal Force Measurements

Climbers achieved higher maximal forces in the half-crimp compared to the open-hand grip position on a 25 mm edge, regardless of age group. This outcome is supported with the biomechanical analysis of Schweizer et al. (2001), who demonstrated that the crimp grip position enables the generation of greater grip forces but also results in increased stress on the finger pulley system [43]. However, Zihlmann et al. (2025) observed comparable forces between the half-crimp and open-hand grip positions on a 23 mm edge in a cross-sectional study of climbers of different performance levels [38], whereas Winkler et al. [47] found the half-crimp superior on an 8 mm edge. These discrepancies may arise from different climbing hold characteristics as used in this study with round shaped edges as well as biases such as climber’s preferences, training history, and neuromuscular adaptations. Moreover, neither study quantified joint angles in order to standardize grip execution. In our study, no full crimp was used during maximal force task, which would have led to higher force results. If we compare the observed half-crimp and open-hand positions during execution of maximal force with the grip during the climbing task, we found a less flexed PIP and less hyperextended DIP, concluding that even a more open grip position was used compared to the climbing tasks.

The analysis of maximal strength was limited to male climbers because of the predominant male cohort and the well-established sex-related differences in maximal finger flexor strength among climbers [35]. Consequently, the present findings regarding maximal finger strength should be interpreted as applicable to only male climbers and cannot be generalized to female climbers.

The angular differences between the maximal force measurements and the climbing tasks could be caused by the loading conditions under which the assessments were performed. During climbing, finger forces are rarely brought to their maximal capacity, as load is distributed to footholds, and other grip positions reduce peak loading of the fingers. The maximal force test imposed a unidirectional load directly on the fingers under eccentric loading conditions, without any distribution of force through the footholds. This might produce a passive extension and an opening torque at the PIP joints. In contrast, the DIP joints showed positive angular differences, indicating the joints to be more flexed compared to the climbing tasks. Other reasons for angular differences in all evaluated joints could be the different body position compared to the body position during the climbing tasks, as body position and the center of mass affect force application and joint angles [45]. In addition, fatigue of the forearm muscles may have contributed to a progressive opening of the grip position.

While a more open-hand grip position in maximal force task was used, it could assume that less injuries of the flexor-tendon-pulley system will be generated. However, pulley injuries are even more seen on hang-boarding, training maximal force, than in rock climbing [9,46]. Schöffl et al. [47] demonstrated in a previous study that lower forces are required to cause pulley injuries under eccentric loading conditions. As hang-boarding leads to over-use training, tiring the forearm muscle and resulting in opening the hand grip as well as producing a passive opening torque, it generates an eccentric force, and injuries could arise more often.

### 4.5. Limitations and Outlook

The numbers of participants were small and predominantly male, with only two female climbers included, which limits the generalizability of the findings. Given the known substantial sex-related differences in strength, the analysis of grip force was restricted to male participants. Therefore, the observed patterns of PIP flexion differences between age groups and their relationship with osteophytes need to be verified for in female climbers.

The extensive measurements generated a large volume of data requiring analysis. Given the hypothesis-driven design and the interdependence of finger joint angles (e.g., a more open grip position involves changes in all the finger joints), no formal correction for multiple testing was applied. Importantly, the main findings showed large group differences and remained highly significant despite the small sample size. Nevertheless, findings with *p*-values close to the significance threshold should be considered exploratory until they are confirmed in future, preferably longitudinal, studies.

Finally, only the handholds were instrumented, and no force measurements were obtained from the feet. Consequently, the distribution of forces between the hands and feet could not be assessed. Future studies incorporating force measurements at the footholds are needed to determine whether older climbers generally exert lower forces during climbing (e.g., due to a less dynamic climbing style) or use different load-distribution strategies, as well as to investigate how differences in foot usage affect finger joint loading and overall climbing technique.

## 5. Conclusions

In summary, our results show that older climbers adopt less extreme finger joint positions during climbing, characterized by significantly reduced flexion of the proximal interphalangeal (PIP) joints and less pronounced hyperextension of the distal interphalangeal (DIP) joints, particularly in the crimp, half-crimp, and self-selected grip positions. These kinematic differences are associated with a higher prevalence of osteophytes, without a reduction in maximum grip strength. However, these findings were limited to male climbers. Notably, climbers of all ages did not use their full finger range of motion during climbing. These findings suggest that long-term exposure to high mechanical loads in extreme joint positions, accompanied by the development of degenerative changes, may lead older climbers to progressively adopt more joint-protective climbing patterns. Although these kinematic adaptations occurred without a loss in maximal grip strength, these movement patterns may also be relevant for younger climbers as a potential strategy for reducing long-term degenerative joint changes and preventing injury by protecting the flexor tendon-pulley system under high loads while reducing joint angulation. Furthermore, older climbers applied less force through their fingers during the actual climbing task, potentially enhancing the joint-protective effect.

Future longitudinal studies are required to clarify the temporal relationships among osteophyte development, changes in finger joint kinematics and the distribution of load between the hands and feet during climbing. Such studies may also help determine whether these adaptations represent protective mechanisms or progressive consequences of cumulative climbing exposure. A better understanding of age-related adaptations may support the development of targeted preventive strategies.

## Figures and Tables

**Table 1:** Anthropometric data, climbing experience (years), training hours per week (h) and max redpoint (RP) bouldering as well as lead climbing level according to IRCRA [34] of the participants. Mean values and standard deviation (SD) of all climbers. CLIM_0028 was excluded as the instrumented grips were not working properly.

Participant	Sex	Age	Weight	Height	Climbing Experience	Training Hours per Week	Max RP Bouldering IRCRA	Max RP Lead Climbing IRCRA	Exclusions/Missing Data
		(Years)	(kg)	(cm)	(Years)	(h)	(Max)	(Max)	
CLIM_0020	M	58	69	180	27	7	26.5	23	no kinematics during maximum force trials
CLIM_0021	M	41	75	177	25	9	28.5	26	no maximum force trials
CLIM_0022	M	34	73	185	11	12	24.5	21	
CLIM_0023	F	31	69	168	23	12	27.5	24	Females excluded for force assessment
CLIM_0024	M	26	75	176	13	11	28.5	24	
CLIM_0025	M	37	66	174	18	4	26.5	24	
CLIM_0026	M	38	65	174	19	10	28.5	29	
CLIM_0027	M	53	81	167	36	6	28.5	26	
CLIM_0029	M	44	74	174	18	3	26.5	19	
CLIM_0030	M	29	73	179	12	9	27.5	21	
CLIM_0031	M	25	72	188	18	30	32.5	29	
CLIM_0032	M	53	81	183	40	8	26.5	27	
CLIM_0033	M	62	71	179	50	4	26.5	26	
CLIM_0034	M	50	74	181	33	4	24.5	24	
CLIM_0035	M	60	71	182	47	10	27.5	27	
CLIM_0036	M	59	86	182	46	3	27.5	26	
CLIM_0037	M	52	67	179	32	5	27.5	28	
CLIM_0038	M	51	77	190	32	7	24.5	24	
CLIM_0039	F	52	56	165	32	12	25.5	23	Females excluded for force assessment
CLIM_0040	M	64	71	180	47	15	22.5	26	
Mean	19M 2F	46	72.3	178.2	29	9.1	26.9	24.9	
SD		12.2	6.3	6.3	12.2	5.9	2.0	2.6	

**Table 2:** Mean and standard deviation (SD) of anthropometric data, climbing experience (years), training hours per week (h) and max redpoint (RP) bouldering as well as lead climbing level according to IRCRA [31] classified in the young and old group of the participants.

Group	Count	Age	Weight	Height	Climbing Experience	Training Hours per Week	Max RP Bouldering IRCRA	Max RP Lead Climbing IRCRA
		(Years)	(kg)	(cm)	(Years)	(h)	(Max)	(Max)
Young	9 (8 male, 1 female)	33.9	71.3	177.2	17.4	11.1	27.8	24.1
SD		6.3	3.6	5.7	4.5	7.3	2.1	3.3
Old	11 (10 male, 1 female)	55.8	73.1	179	38.4	7.4	26.1	25.5
SD		4.7	7.8	6.6	7.6	3.5	1.7	1.6

**Table 3:** Mean joint angle of left and right PIP 3 and 4 as well as DIP 3 and 4 and standard deviation (SD) (in °) of older (*n* = 11) and younger (*n* = 9) climbers in crimp, half-crimp, open-hand grips and self-selected grip positions during the holding phase (P2). *p*-value < 0.05 denote significant differences.

Task	Joint	Old Climbers Mean (SD) [°]	Young Climbers Mean (SD) [°]	*p*-Value
Crimp	PIP	65.0 (17.0)	85.1 (11.4)	<0.0001
	DIP	−2.2 (11.8)	−15.7 (10.2)	<0.0001
Half-Crimp	PIP	52.6 (16.7)	74.9 (12.5)	<0.0001
	DIP	7.2 (12.2)	−6.7 (9.2)	<0.0001
Open-Hand Grip	PIP	33.0 (17.1)	36.7 (24.4)	0.44
	DIP	18.4 (11.5)	18.5 (14.4)	0.83
Self-Selected	PIP	55.7 (17.7)	75.6 (10.2)	<0.0001
	DIP	5.1 (12.4)	−6.8 (7.7)	<0.0001

**Table 4:** Mean joint extension, flexion and ROM and standard deviation (SD) of left and right PIP, DIP as well as MCP of the middle and ring finger and the wrist (in °) of old (*n* = 11) and young (*n* = 9) climbers during basic motion task. *p*-value < 0.05 denote significant differences.

Joint	Movement	Old Climbers Mean (SD) [°]	Young Climbers Mean (SD) [°]	*p*-Value
DIP	Extension	−4.7 (3.6)	−5.6 (3.1)	0.323
	Flexion	62.2 (12.2)	65.2 (4.9)	0.879
	ROM	66.8 (10.1)	70.8 (4.9)	0.649
PIP	Extension	−5.2 (5.1)	−8.6 (4.4)	0.153
	Flexion	86.6 (15.3)	112.3 (6.0)	0.0003
	ROM	91.8 (18.3)	120.9 (7.4)	0.002
MCP	Extension	−12.5 (7.5)	−8.3 (3.3)	0.135
	Flexion	97.4 (9.8)	94.8 (6.0)	0.507
	ROM	109.9 (10.3)	103.1 (8.4)	0.145
wrist	Extension	−70.3 (4.3)	−78.1 (4.8)	0.003
	Flexion	63.0 (7.7)	61.2 (7.1)	0.630
	ROM	133.3 (8.9)	139.3 (7.3)	0.157

**Table 5:** Mean and standard deviation (SD) of grip force during the holding phase (P2) of older (*n* = 10) and younger (*n* = 8) male climbers in crimp, half-crimp, open-hand grips and self-selected grip reported in % body weight. *p*-value < 0.05 denote significant differences.

Task	Old Climbers Mean (SD) [BW]	Young Climbers Mean (SD) [BW]	*p*-Value
Crimp	39.7% (2.2%)	42.9% (3.0%)	0.030
Half-Crimp	40.6% (2.5%)	45.5% (2.5%)	0.001
Open-Hand Grip	40.0% (2.1%)	43.5% (2.5%)	0.009
Self-Selected	41.7% (1.8%)	45.6% (2.1%)	0.001

**Table 6:** Mean difference in PIP and DIP joint angle (in °) between JAMF and JACT in the grip positions half-crimp and open-hand executed with the dominant hand (DH) and non-dominant hand (NDH) hand (old *n* = 9; young *n* = 7; total *n* = 16). Positive angular differences indicate that the joint was more flexed, while negative differences suggest less flexion compared to the climbing tasks.

Task	DH/NDH	PIP3		PIP4		DIP3		DIP4	
		Mean (°)	SD (°)	Mean (°)	SD (°)	Mean (°)	SD (°)	Mean (°)	SD (°)
Half-crimp	DH	0.4	9.5	−5.6	13.4	15.9	10.8	23.1	11.2
Half-crimp	NDH	0.9	11.4	−5.5	12.7	13.7	12.0	18.3	12.0
Open-hand	DH	−7.3	14.4	−14.7	16.7	22.3	12.2	25.4	11.8
Open-hand	NDH	−7.9	10.0	−12.0	15.0	22.7	12.0	19.4	12.0

## Data Availability

The datasets presented in this article are not readily available because the data are part of an ongoing study.

## Abbreviations

The following abbreviations are used in this manuscript:

BW	body weight
DH	dominant hand
DIP	distal interphalangeal joint
e.g.,	for example
FDP	flexor digitorum profundus
FDS	flexor digitorum superficialis
Fres	resultant force
h	hours
HC	half-crimp
IRCRA	international rock climbing research association
JAMF	joint angle at maximal force
JACT	mean joint angle during the holding phase (only abbreviated for maximal force task for simplified representation)
kg	kilogramm
MCP	metacarpophalangeal joint
mm	millimeters
ms	milliseconds
N	Newton
NDH	non-dominant hand
O	open-hand
P1	gripping phase
P2	holding phase
P3	releasing phase
PIP	proximal interphalangeal joint
RP	redpoint
SD	standard deviation
*	significant difference

## Appendix A

**Table A1:** Correlation between age and PIP Flexion during climbing for the different grip conditions.

Grip Condition	R	*p*-Value
Crimp	−0.540	0.014
Half-Crimp	−0.598	0.005
Open-Hand Grip	0.104	0.663
Self-Selected	−0.510	0.021

**Table A2:** Correlation between age and maximum finger ROM. (* show significant differences).

Gelenk	p_Extension	R_Extension	p_Flexion	R_Flexion	p_ROM	R_ROM
PIP	0.283	0.252	0.001 *	−0.673	0.017 *	−0.526
DIP	0.945	−0.017	0.316	−0.236	0.204	−0.297
MCP	0.057	−0.433	0.769	0.070	0.252	0.269
wrist	0.001 *	0.689	0.561	0.142	0.112	−0.377
